# Efficacy of duloxetine in treating temporomandibular joint disorder: a systematic review with bayesian meta-analysis

**DOI:** 10.1186/s12903-025-06043-w

**Published:** 2025-08-07

**Authors:** Sasidharan Sivakumar, Ashok K. Sundramoorthy, Sankar Narayanan, Julia Thenmozhi J, Gowardhan Sivakumar

**Affiliations:** 1 Discovery Research Division, Indian Council of Medical Research, ICMR Headquarters, New Delhi, 110029 India; 2 Department of Public Health Dentistry, Saveetha Institute of Medical and Technical Sciences, Tamil Nadu, 600077 India; 3 Department of Maxillofacial Prosthodontics, Saveetha Institute of Medical and Technical Sciences, Tamil Nadu, 600077 India; 4 Department of Oral Medicine & Radiology, Sree Balaji Dental College & Hospital, Tamil Nadu, 600100 India; 5 Department of Oral & Maxillofacial Pathology, KLE VK Institute of Dental sciences, Karnataka, 590010 India; 6 Department of Maxillofacial Prosthodontics, Ragas Dental College and Hospital, Tamil Nadu, 600119 India

**Keywords:** Temporomandibular joint disorders, Arthrocentesis, Duloxetine, Serotonin, Bayesian analysis

## Abstract

**Objectives:**

This study aims to evaluate the efficacy of duloxetine in managing temporomandibular joint disorder (TMD), focusing on pain reduction and functional improvement. The research addresses the need for novel therapeutic agents due to the suboptimal efficacy and tolerability of existing treatments.

**Materials and Methods:**

A systematic review and Bayesian meta-analysis were conducted following PRISMA guidelines. A comprehensive search of MEDLINE (via PubMed), Web of Science, Scopus, Embase, and Google Scholar was performed up to February 2025. Eligibility criteria were defined using the PICOS framework: Population (TMD patients), Intervention (duloxetine), Comparator (placebo or other treatments), Outcomes (pain reduction, maximum mouth opening), and Study design (randomized controlled trials). Data synthesis employed Bayesian meta-analysis, and evidence quality was assessed using the GRADE framework.

**Results:**

Five studies involving 203 participants met the inclusion criteria. Four evaluated duloxetine combined with TMJ arthrocentesis, while one compared duloxetine to a placebo. Combination therapy yielded significant pain reduction (pooled effect size = 1.42) and a consistent, though not statistically significant, improvement in maximum mouth opening. Bayesian analysis strongly supported pain reduction (BF_10_ = 44.197) but was inconclusive for functional improvement (BF_10_ = 0.783). The risk of bias ranged from moderate to high, with high-certainty evidence supporting the efficacy of combination therapy.

**Conclusion:**

Duloxetine, when combined with TMJ arthrocentesis, provides significant pain relief and potential functional benefits in TMD management. However, further large-scale, high-quality randomized trials are necessary to confirm these findings.

## Introduction

Temporomandibular Joint Disorder (TMD) represents a complex and multifaceted condition affecting the temporomandibular joint, muscles of mastication, and associated structures. It encompasses a spectrum of symptoms including jaw pain, restricted mandibular movement, clicking or popping sounds during jaw movement, muscle tenderness, and even headaches. In recent decades, the prevalence of TMD has become increasingly notable, encompassing a significant proportion of both adult and paediatric populations. Studies indicate an overall prevalence rate of approximately 31% among adults and elderly individuals, with a corresponding rate of 11% observed among children and adolescents. Notably, the most prevalent form of TMD is characterized by disc displacement with reduction [1]. Gender disparities are also evident, with women exhibiting a markedly higher prevalence rate, ranging from two to four times that of men. This gender discrepancy reaches its peak between the ages of 25 and 45, highlighting a critical period of vulnerability for TMD onset and manifestation [2]. Recent studies have shed light on its substantial impact on quality of life, with patients often experiencing chronic pain, functional impairment, and psychological distress [3]. Furthermore, TMJ disorder has been linked to comorbidities such as sleep disturbances and depression, highlighting its far-reaching consequences.

Current management strategies for TMD encompass a diverse range of approaches, including pharmacotherapy with non-steroidal anti-inflammatory drugs (NSAIDs) and nutraceutical agents such as glucosamine hydrochloride [4], physical therapy, occlusal splint therapy [5], arthrocentesis [6], and surgical interventions. However, their efficacy and tolerability remain suboptimal for a significant number of patients, highlighting the urgent need for novel therapeutic agents specifically designed for TMJ disorder [7]. Recent research has identified promising targets for drug development, including agents that modulate neuroinflammation, regulate pain signalling pathways, and promote tissue repair within the temporomandibular joint.

Duloxetine, a serotonin-norepinephrine reuptake inhibitor (SNRI), has emerged as a promising pharmacological option for treating TMDs, particularly in cases where pain and mood disturbances coexist [8]. Recent studies have highlighted duloxetine's efficacy in alleviating both pain and associated psychological symptoms commonly observed in TMJ disorder patients [9]. Furthermore, duloxetine's dual mechanism of action, targeting both serotonin and norepinephrine reuptake, may confer additional benefits in addressing the complex neurobiological underpinnings of TMJ disorder, which involve aberrant pain processing and mood dysregulation [10, 11]. Notably, duloxetine's efficacy in managing chronic pain conditions with a neuropathic component, such as fibromyalgia [12], lends support to its potential utility in TMJ disorder management.

This systematic review aims to critically evaluate the efficacy of duloxetine in treating TMJ disorders through a comprehensive analysis of randomized controlled trials (RCTs). By employing Bayesian meta-analysis, we intend to provide a meticulous synthesis of the available data, accounting for both the magnitude and uncertainty of treatment effects. Additionally, utilized the Grading of Recommendations, Assessment, Development, and Evaluations (GRADE) framework to assess the quality of evidence, ensuring a rigorous evaluation of the findings. Our review seeks to clarify the therapeutic value of duloxetine for TMJ disorders, offering insights that may guide clinical decision-making and future research endeavours.

## Materials and methods

### Review question

This systematic review was conducted following the “Preferred Reporting Items for Systematic Reviews and Meta-Analyses” (PRISMA) guidelines [13], to answer the following research question: “What is the efficacy of Duloxetine in reducing pain and improving functional outcomes in patients with Temporomandibular Joint Disorder compared to standard treatment modalities?”.

### PICOS criteria

The eligibility criteria were decided considering the following PICOS definition:

- **(P) Population:** Subjects with temporomandibular joint disorders
- **(I) Intervention:** Subjects treated with duloxetine
- **(C) Comparison:** Subjects treated with placebo or any other treatment modality
- **(O) Outcomes:** Reduction in pain, increase in maximum mouth opening and reduction in clicking or popping sound
- **(S) Study design:** Experimental trial

### Inclusion and exclusion criteria

The inclusion criteria encompassed original experimental trials that investigated the effects of duloxetine in managing TMDs. Studies were required to include subjects diagnosed with TMDs, where duloxetine was administered as an intervention, with a comparison group receiving either a placebo or an alternative treatment modality. Eligible studies had to report on at least one primary outcome, such as pain reduction, improvement in maximum mouth opening, or a decrease in clicking or popping sounds. Only studies adhering to the PICO framework were considered to ensure methodological consistency.

Exclusion criteria comprised case reports, case series, review articles, editorials, and non-experimental studies, as they do not provide robust comparative evidence. Studies lacking a control group or failing to assess the defined primary outcomes were also excluded. Furthermore, trials involving participants with confounding conditions that could influence TMD symptoms, such as concurrent neurological or systemic disorders, were omitted to maintain the validity of the findings.

### Search strategy for article identification

Two independent researchers (S.S and G.S) performed an electronic search for randomized clinical trials addressing the research question across the databases: MEDLINE (via PubMed), Web of Science, Scopus, Embase, and Google Scholar, up to February, 2025. The detailed search strategies employed for each databases are outlined in Table 1. The search was restricted to publications in English, with no additional restriction criteria applied.

**Table 1:** Search strategy for the databases

Database	Nos	Search strategy
MEDLINE (PubMed)	2	(((((((((diseases, tmj[MeSH Terms]) OR (disease, tmj[MeSH Terms])) OR (disorder, tmj[MeSH Terms])) OR (disorders, tmj[MeSH Terms])) OR (syndrome, tmj[MeSH Terms])) OR (temporomandibular joint disease[MeSH Terms])) OR (temporomandibular joint diseases[MeSH Terms])) OR (temporomandibular joint disorder[MeSH Terms])) OR (temporomandibular joint disorders[MeSH Terms])) AND (duloxetine hydrochloride[MeSH Terms])
Scopus	56	((TITLE-ABS-KEY ("temporomandibular joint disorders") OR TITLE-ABS-KEY ("tmj disorder") OR TITLE-ABS-KEY (temporomandibular) OR TITLE-ABS-KEY (tmj) OR TITLE-ABS-KEY ("tmj diseases"))) AND (TITLE-ABS-KEY (duloxetine))
Web of Science	8	(((TS = (temporomandibular joint disorders)) OR TS = (tmj disorders)) OR TS = (temporomandibular)) OR TS = (tmj disease)) AND TS = (duloxetine)
Embase	39	('temporomandibular joint disorder'/exp OR'costen syndrome'OR'craniomandibular disorders'OR'craniomandibular joint syndrome'OR'temporomandibular dysfunction'OR'temporomandibular joint disease'OR'temporomandibular joint diseases'OR'temporomandibular joint disorder'OR'temporomandibular joint disorders'OR'temporomandibular joint dysfunction'OR'temporomandibular joint dysfunction syndrome'OR'temporomandibular joint pain'OR'temporomandibular joint syndrome') AND ('duloxetine'/exp OR'duloxetine hydrochloride')
Google scholar	11	"Duloxetine"AND"Temporomandibular Joint Disorders"OR"Temporomandibular Joint Dysfunction"

### Screening of articles

**Fig. 1:**
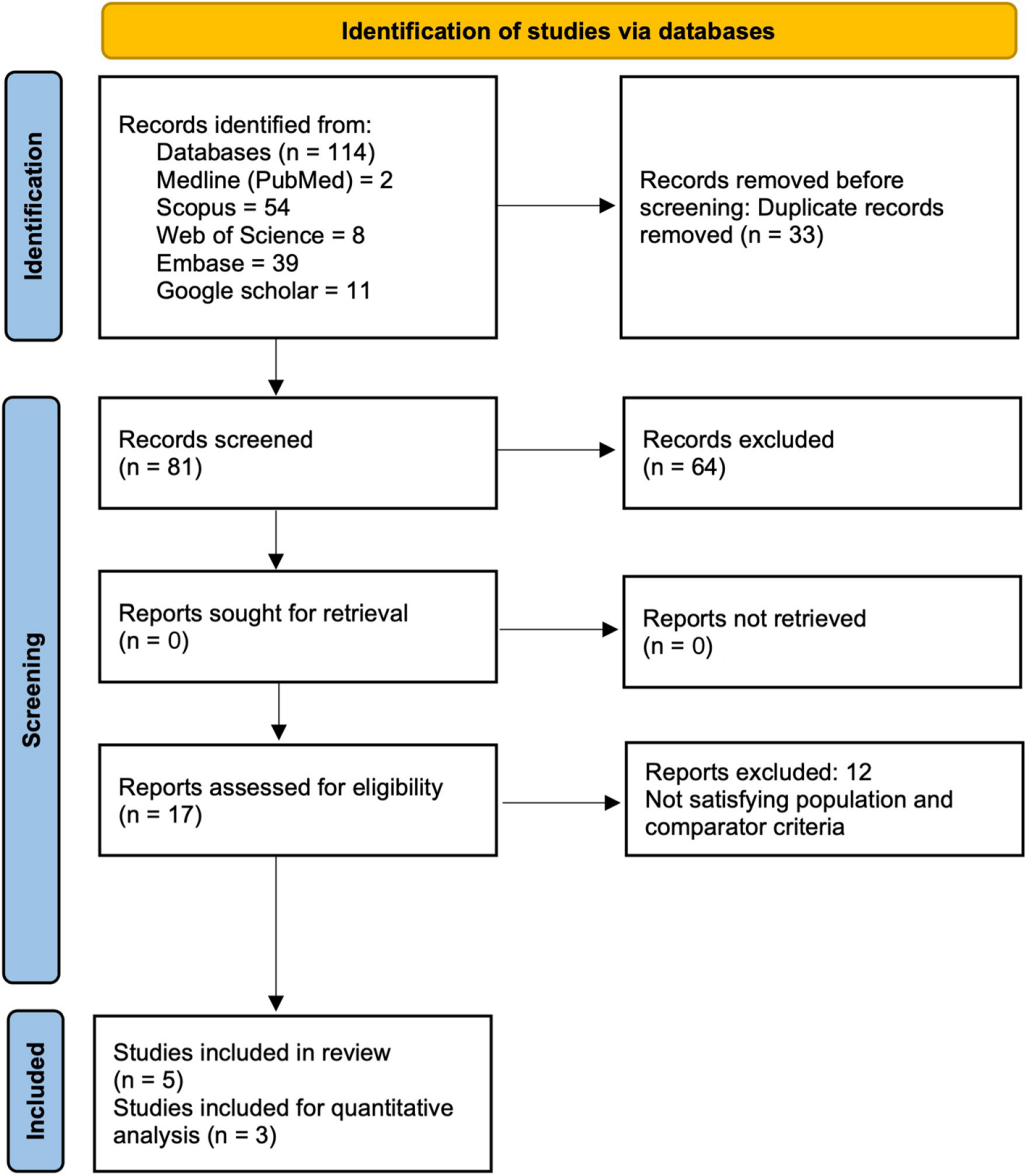
The PRISMA Flow chart

After pooling articles from various databases, duplicates were identified and removed based on similarities in title, author, and year of publication. Two authors (S.S and G.S) independently conducted the initial screening of titles and abstracts. Studies that did not meet the specified PICO criteria were excluded upon mutual agreement. Any disagreements regarding article selection were resolved through discussion and, if necessary, consultation with a third author (A.S). Subsequently, the full texts of eligible articles were reviewed individually by the same two authors (S.S and G.S), with any discrepancies resolved through further discussion (Fig. 1). The level of agreement calculated through Cohen’s kappa between the two authors (S.S and G.S) was 0.90 for titles and abstracts and 0.94 for full texts.

### Data extraction

For all studies meeting the eligibility criteria, data extraction was conducted independently by two authors (S.S and G.S). The extracted data included the following parameters: (a) Author and year of publication, (b) Type of study, (c) Study location, (d) Data location, (e) Characteristics of the study group, (f) Characteristics of the control group, (g) Arthrocentesis rinsing solution, (h) Evaluation parameters, (i) Evaluation period, (j) Results in the test group, (k) Results in the control group, (l) Statistical significance between groups, and (m) Overall inference. The extracted data were organized and presented in Tables 2 and 3.

**Table 2:** General characteristics of included studies

Author, year	Type of study	Study location	Data location	Study group (n)	Control group (n)	Arthrocentesis rinsing solution	Evaluation parameter	Period of evaluation
Singh RK et al., 2017 [17]	Randomized clinical trial	India	Figure 2	TMJ arthrocentesis with duloxetine 30 mg BD (10)	TMJ arthrocentesis (10)	Normal saline	Pain- Graded chronic pain scale Maximal mouth opening- millimetres Clicking sound- Present/absent Hospital Anxiety and Depression Rating Scale Interleukin 6	12 weeks
Goyal P et al., 2020 [9]	Clinical trial	India	Tables 1 & 2	**Group B** – Duloxetine 30 mg BD (10) **Group C**—TMJ arthrocentesis with Duloxetine 30 mg BD (10)	**Group A**—TMJ arthrocentesis (10)	Ringer’s lactate solution	Pain- VAS Maximal mouth opening- millimetres Clicking sound- Present/absent Hospital Anxiety and Depression Rating Scale Interleukin 6	12 weeks
Mamit Kumar DS et al., 2021 [22]	Clinical trial	India	Tables 1, 2, & 3	**Group B** – Duloxetine 30 mg BD (15) **Group C**—TMJ arthrocentesis with Duloxetine 30 mg BD (15)	**Group A**—TMJ arthrocentesis (15)	Ringer’s lactate solution	Pain- VAS Maximal mouth opening- millimetres Clicking sound- Present/absent Interleukin 6	12 weeks
Abdallah AMA et al., 2024 [21]	Randomized clinical trial	Egypt	Figure 2	TMJ arthrocentesis with duloxetine 30 mg BD (14)	TMJ arthrocentesis (14)	Ringer’s lactate solution	Pain- VAS Maximum mouth opening (MMO)- millimetres Anxiety and depression: Patient health questionnaire-9	12 weeks with 6 months observation
Ferreira D.M.A.O.; et al., 2024 [16]	Randomised, placebo-controlled clinical trial	Brazil	Table 2	Duloxetine 30 mg BD with Self-management (SM) strategy (40)	Placebo with SM strategy (40)	NA	Pain (NRS) Pittsburgh Sleep Quality Index Hospital Anxiety and Depression Scale -to assess anxiety and depression	12 weeks

*NA* Not applicable

**Table 3:** Outcome characteristics of included studies

Author, year	Variables	Results (Mean(SD))		Significance	Inference
		**Test group**	**Control group**		
Singh RK et al., 2017 [17]	**Pain**	4.3	1.9	0.001*	Combination of duloxetine with arthrocentesis gave much better outcome than arthrocentesis alone in the primary outcome measures such as pain and maximal mouth opening
	**MMO**^b^	−8.0	**-**3.0	0.001*	
	**Clicking sound** ^**†**^			insignificant	
	**Interleukin 6**	Values not available		insignificant	
Goyal P et al., 2020 [9]	**Pain**	**GB** (0.5 (2.41)) **GC** (4.31 (0.78))	2.0 (1.98)	0.001*	Group C subjects experienced significantly less pain and improved mouth opening compared to Groups A and B at specific intervals. Additionally, a marked reduction in IL-6 levels was observed postoperatively in Groups A and C
	**MMO**^b^	**GB** (−0.6 (5.67)) **GC** (−7.0 (9.40))	−3.20(7.54)	0.001*	
	**Clicking sound**^a^			insignificant	
	**Interleukin 6**	**GC** (4.28 (5.03))	6.85 (6.83)	insignificant	
Mamit Kumar DS et al., 2021 [22]	**Pain**	**GB** (0.52 (2.18)) **GC** (4.31 (1.17))	2.01 (0.97)	0.001*	Combining arthrocentesis with duloxetine significantly reduces pain and improves maximum mouth opening more effectively and rapidly than either treatment alone
	**MMO**^b^	**GB** (−0.36 (6.45)) **GC** (−7.02 (9.36))	−3.18(7.57)	0.001*	
	**Clicking sound**^a^			insignificant	
	**Interleukin 6**	GC (4.33 (5.57))	6.74 (6.9)	Insignificant	
Abdallah AMA et al., 2024 [21]	**Pain**	6.14 (1.10)	5.00 (1.11)	0.029*	Combining duloxetine with arthrocentesis can offer effective and sustained pain relief. However, this approach carries an increased risk of adverse events, and the safety and efficacy of duloxetine for durations exceeding three months require further investigation
	**MMO**^b^	−11.14 (1.41)	−10.50 (2.95)	insignificant	
	**PHQ 9**	6.57 (2.47)	5.93 (2.92)	insignificant	
Ferreira D.M.A.O.; et al., 2024 [16]	**Pain**	−2.1 (3.36)	−2.4 (2.93)	0.820	This study did not demonstrate any additional benefits of incorporating duloxetine into standard self-management strategies for treating painful TMD
	**PSQI**	−0.6 (3.7)	−2.8 (2.7)	0.003*	
	**HADS Anxiety**	−0.7 (2.5)	−1.4 (2.8)	insignificant	
	**HADS Depression**	−0.1 (2.2)	−0.6 (2.8)	insignificant	

*p value of≤0.05 is considered significant

^a^Qualitative output

^b^(−) minus mark indicates increase in MMO (maximum mouth opening) parameter

### Risk of Bias

Each article was critically evaluated independently by two reviewers using the Cochrane Risk of Bias Tool version 2.0 for randomized controlled trials [14]. This tool encompasses five domains of assessment that collectively determine the overall risk of bias. Each domain contains a specific set of questions, with responses categorized as “yes,” “no,” “probably yes,” “probably no,” and “not applicable.” A summary score was generated for each study based on its adherence to quality criteria and fulfilment of requirements. Any disagreements between the two reviewers were resolved by consultation with a third reviewer (A.S.).

#### Data preparation for comparable evidence synthesis

To facilitate the quantitative evaluation of the pooled study results, a conservative approach was employed to estimate the difference in standard deviations from baseline in studies where the standard deviation of the mean difference between baseline and post-treatment was not directly available. This was calculated using the following formula:

$$\mathbf{S}\mathbf{D}(\mathbf{c}\mathbf{h}\mathbf{a}\mathbf{n}\mathbf{g}\mathbf{e}\mathbf{f}\mathbf{r}\mathbf{o}\mathbf{m}\mathbf{b}\mathbf{a}\mathbf{s}\mathbf{e}\mathbf{l}\mathbf{i}\mathbf{n}\mathbf{e})=\sqrt{{\mathbf{S}\mathbf{D}\mathbf{p}\mathbf{r}\mathbf{e}}^{2}+{\mathbf{S}\mathbf{D}\mathbf{p}\mathbf{o}\mathbf{s}\mathbf{t}}^{2}-2.\mathbf{r}.\mathbf{S}\mathbf{D}\mathbf{p}\mathbf{r}\mathbf{e}.\mathbf{S}\mathbf{D}\mathbf{p}\mathbf{o}\mathbf{s}\mathbf{t}}$$

Here, the correlation coefficient (r) was estimated using the mean and standard deviation values of the groups, with calculations performed using the Campbell Collaboration effect size calculator [15].

To ensure consistency and standardization across the included studies, we addressed the absence of the standard deviation for the mean in the study by Ferreira, D.M.A.O., et al., 2024 [16]. This study provided the mean and 95% confidence interval for its evaluation parameter. We harmonized this data with that of other studies by deriving the standard deviation (σ) from the provided confidence interval using the following formula:

$$\mathbf{S}\mathbf{t}\mathbf{a}\mathbf{n}\mathbf{d}\mathbf{a}\mathbf{r}\mathbf{d}\mathbf{d}\mathbf{e}\mathbf{v}\mathbf{i}\mathbf{a}\mathbf{t}\mathbf{i}\mathbf{o}\mathbf{n}({\varvec{\upsigma}})=(\mathbf{M}\mathbf{E}.\sqrt{{\varvec{n}})/\mathbf{z}}$$

where,

ME- margin of error, calculated as half the width of the confidence interval,

n- sample size,

z- z-score corresponding to the 95% confidence level (approximately 1.96).

These estimated quantitative values are presented in Table 3. However, in the study by Singh RK et al., [17] the standard deviations for the mean in their pain or mouth opening parameters were not mentioned, thereby limiting their application for generating quantitative forest plot graphs. By employing these methods, we aimed to enhance the robustness and comparability of the quantitative analysis across the included studies.

### Effect measure and Statistical analysis

The comparable data from the included studies were entered into Microsoft Excel 2016. Bayesian meta-analysis was conducted using JASP software [18] on selected studies with comparable outcomes from systematically reviewed articles. Despite the low heterogeneity among the studies, the small sample sizes and limited number of studies necessitated the use of a Bayesian model to enhance the robustness of the evidence. To address this limitation, we employed Markov Chain Monte Carlo (MCMC) methods in Bayesian meta-analysis to estimate effect sizes.

In our analysis, we used non-informative priors to allow the data to primarily drive the posterior estimates. Specifically, the prior for the overall effect size (μ) was set as a diffuse normal distribution N(0,10^2^), while the prior for the between-study variance (τ^2^) followed a vague half-Cauchy or inverse-gamma distribution. By leveraging MCMC, we sampled from the posterior distribution, integrating both prior information and observed data. Consequently, all summary estimates were derived from these samples and compared with observed estimates to strengthen the evidence. Both fixed- and random-effects Bayesian models were used in the analysis, and the final pooled estimate was obtained by averaging the results from both models. This model-averaging approach was chosen to account for potential variations in effect size estimation while maintaining robustness.

Quantitative evidence synthesis focused on the reduction in pain and improvements in maximum mouth opening. Cohen’s d with 95% confidence intervals (CI) was used to summarize the data for each group, evaluating the experience of positive outcomes between the study and control groups. This assessment was further strengthened by adopting the GRADE (Grading of Recommendations, Assessment, Development, and Evaluations) approach, using the online software GRADEpro GDT [19], to analyze the certainty of the evidence.

## Results

### Study selection

The studies were screened by two authors (S.S and G.S) based on the PICO criteria at the title and abstract level and at the full-text level, with 5 articles finally included for the review, and based on the comparable similar outcomes in those studies, 3 were utilized for generating quantitative evidence synthesis in the form of Bayesian meta-analysis forest plot graph. The article selection was graphically represented with the PRISMA flow diagram (Fig. 1) [20].

### General characteristics

Our systematic review included a total of five experimental studies [9, 16, 17, 21, 22] conducted between 2017 and 2024, despite no restrictions on publication years. These studies evaluated the efficacy of duloxetine for TMDs, involving a combined total of 203 participants. The primary focus of these studies varied: four studies examined the efficacy of duloxetine in combination with TMJ arthrocentesis [9, 17, 21, 22], while one study investigated duloxetine compared to a placebo along with self-management strategies [16]. The duration of duloxetine administration were almost consistent across all studies, with 12-week of treatment period. Primary outcome measures included reduction in pain, predominantly assessed using the Visual Analogue Scale (VAS). Secondary outcomes included maximal mouth opening measured in millimetres, biomarker estimations, and assessments using various subjective well-being questionnaires.

### Main outcome of the study

The results across the included studies demonstrated that, in the studies combining duloxetine with TMJ arthrocentesis [9, 17, 21, 22], participants showed a marked reduction in pain scores estimated with the VAS and GCPS, indicating the potential benefit of this combined approach. The study comparing duloxetine to a placebo [16] although reported a statistically insignificant reduction in pain yet it favours duloxetine. Across all studies, there was a consistent improvement in maximal mouth opening measurements, suggesting enhanced functional outcomes. Biomarker analysis and subjective well-being assessments further supported these findings, with participants of duloxetine group reporting improved quality of life and reduced symptom severity yet they were insignificant [9, 17, 22]. Overall, the evidence suggests that duloxetine, particularly when used in conjunction with TMJ arthrocentesis, may offer a valuable therapeutic option for managing TMJ disorders. However, the limited number of studies and participants highlights the need for further research to confirm these preliminary findings and better understand the long-term benefits and safety of duloxetine in this context.

### Risk of Bias and quality assessment

**Fig. 2:**
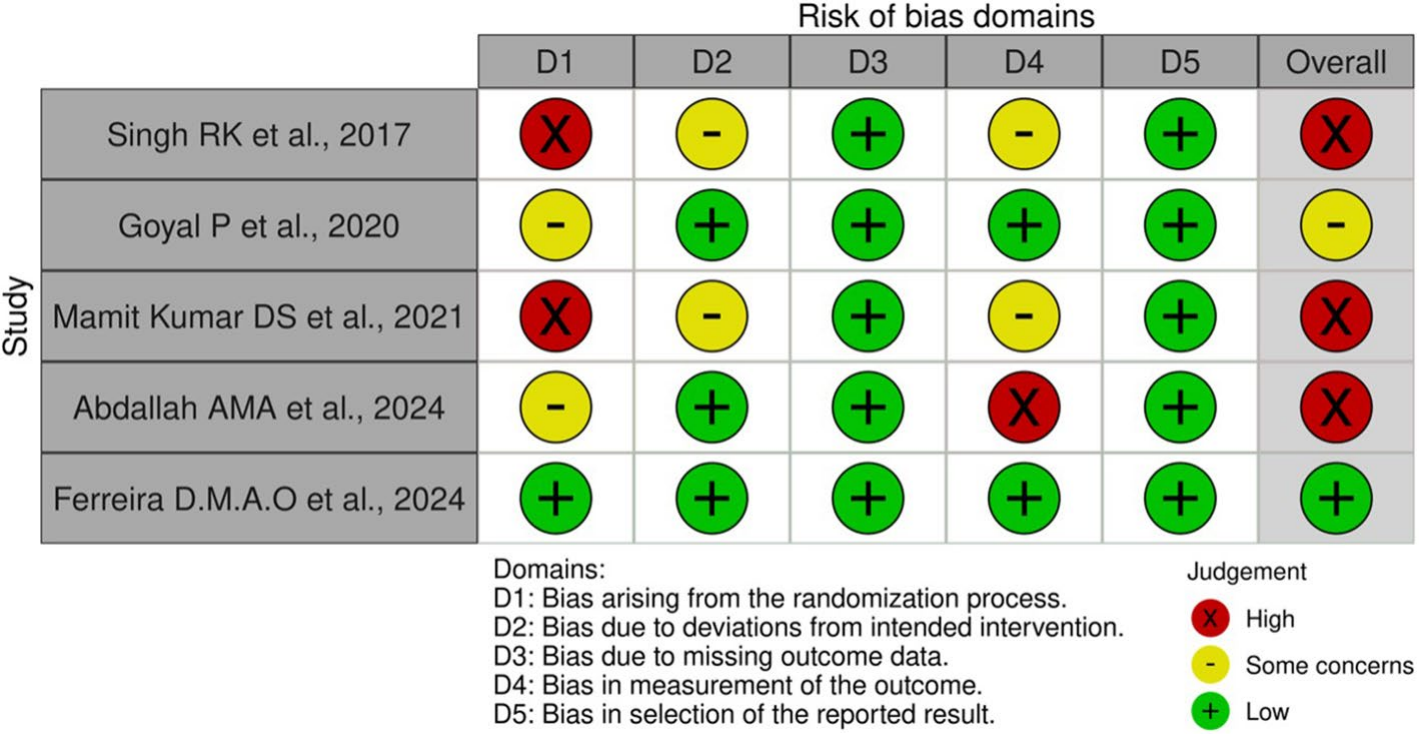
Risk of bias for individual studies

**Fig. 3:**
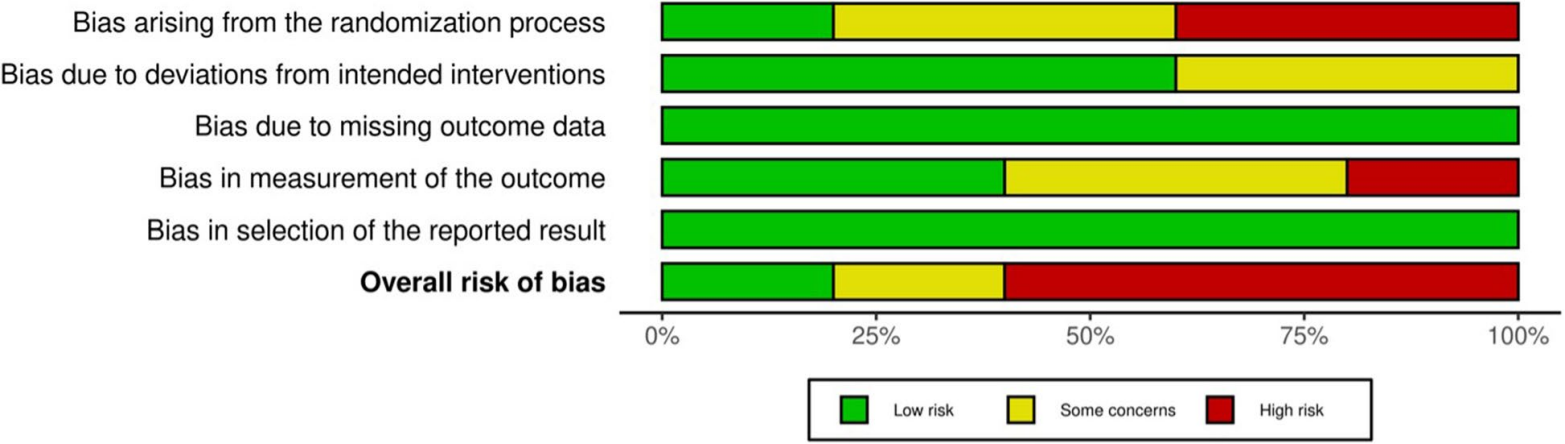
Summary risk of bias graph

The risk of bias in the included studies was assessed using the Cochrane Risk of Bias 2.0 tool [23] for randomized controlled trials, achieving a high interrater reliability with a Kappa score of 0.89. Visualization of the risk of bias for individual studies (Fig. 2) and the overall summary (Fig. 3) revealed that less than 25% of the studies exhibited a low risk of bias. Among the remaining 75%, the studies were equally divided between those at high risk of bias and those with some concerns. Among the assessed studies, only one was rated as having a low risk of bias across all five evaluation domains [16]. The predominant concern was related to the randomization process, contributing significantly to the overall high risk of bias. Performance bias was effectively minimized in two studies through adequate blinding of participants and personnel. However, none of the studies clearly described the blinding of outcome assessment, introducing potential detection bias. Attrition bias was generally low, with minimal loss to follow-up and appropriate explanations for any dropouts. Despite these issues, the overall risk of bias was assessed as high to moderate, suggesting that while the findings should be interpreted with caution, they are generally reliable.

### Quantitative synthesis

A Bayesian model meta-analysis was conducted to evaluate the effect of duloxetine compared to TMJ arthrocentesis and placebo on pain reduction and improvement in maximal mouth opening in patients with temporomandibular joint (TMJ) disorder. In our Bayesian meta-analytic model, effect sizes were estimated using Markov Chain Monte Carlo (MCMC) simulation. These estimated effect sizes were then compared with the observed effect sizes. When the estimated and observed effect sizes closely align, it suggests minimal variability and robustness in the observed values. Conversely, a wide discrepancy between estimated and observed effect sizes indicates significant variation.

### Evaluation of pain parameter

**Fig. 4:**
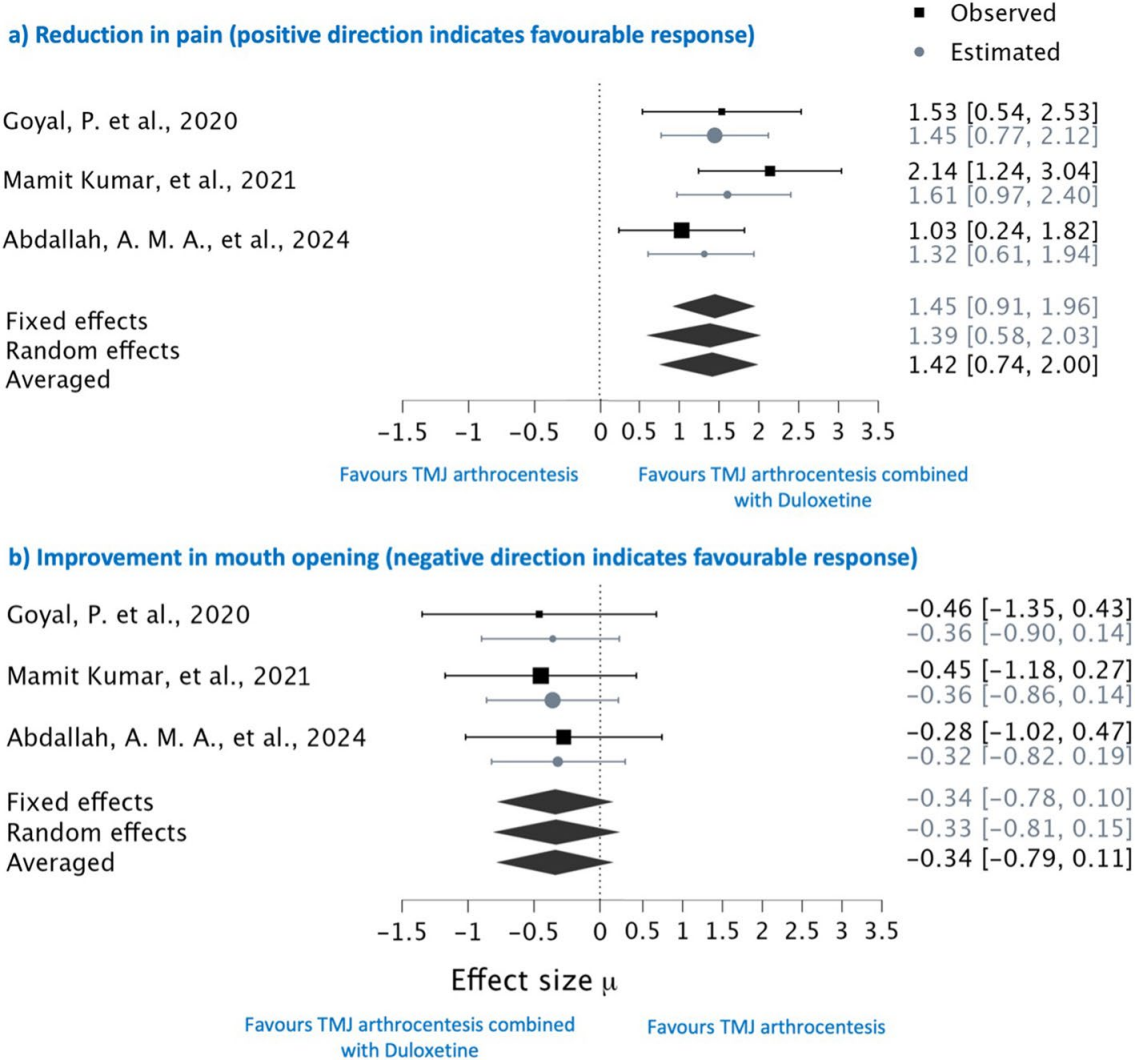
Forest plot graph on efficiency of combining duloxetine with TMJ arthrocentesis in improvising the parameter of (**a**) pain, (**b**) mouth opening

This Bayesian meta-analysis evaluated the efficacy of combining duloxetine with TMJ arthrocentesis compared to standalone TMJ arthrocentesis in reducing pain. Three out of five studies were included in the analysis due to their similarity in interventions. In our analysis, one study comparing the drug duloxetine with a placebo [16] was excluded from our Bayesian meta-analysis. Although it assessed pain reduction, the study was excluded due to dissimilarity in comparators and the limitation of being a single study. Including this study could introduce bias and compromise the reliability of our overall analysis. And another study [17] was excluded due to the absence of standard deviation for their mean values, rendering it unsuitable for quantitative synthesis. Therefore, out of the five studies assessing pain parameters, only three were considered suitable for the Bayesian meta-analysis, ensuring the robustness and accuracy of the generated forest plot graph. The observed effect sizes from the three studies were used to estimate the overall effect size through a Bayesian model. Notably, the observed effect sizes were generally consistent with the estimated values, except for one study which reported extreme values [22]. Additionally, the heterogeneity was considerably low, with an I^2^ value of 21.43%. The Bayesian model effectively moderated these outliers, producing an estimated effect size of 1.614, closer to the overall mean (Fig. 4a), thus demonstrating its capability to adjust for biases and imbalances.

Further, random and fixed effects Bayesian models were employed, yielding a pooled average estimate of 1.42 (Table 4, Fig. 4a), which significantly favoured the combination therapy for reduction in pain for subjects with TMDs. The Bayesian forest plot illustrated that none of the studies lower credible intervals intersected the line of no effect, underscoring the substantial impact of adding duloxetine to TMJ arthrocentesis. This Bayesian meta-analysis robustly supports that duloxetine combined therapy significantly enhances pain reduction compared to standalone TMJ arthrocentesis, offering a compelling recommendation for clinical practice in managing temporomandibular joint disorders.

**Table 4:** Effect sizes per study: observed and estimated values

Author, year	Parameter evaluated	Observed	Estimated^a^		
			**Mean**	**Lower**	**Upper**
Goyal, P. et al., 2020[9]	Pain	1.535	1.446	0.767	2.141
	MMO	−0.463	−0.353	−0.892	0.169
Mamit Kumar, et al., 2021[22]	Pain	2.140	1.614	0.962	2.422
	MMO	−0.451	−0.358	−0.867	0.134
Abdallah, A. M. A., et al., 2024[21]	Pain	1.031	1.306	0.579	1.904
	MMO	−0.277	−0.326	−0.828	0.188

^a^Posterior mean and 95%credible interval estimates from the random effects model

### Evaluation of mouth opening parameter

In the assessment of maximal mouth opening improvement resulting from the intervention of duloxetine combined with TMJ arthrocentesis, three studies were included in the analysis. The effect sizes, denoted by negative signs, indicate that the post-observation data in millimetres exceeded the baseline values, suggesting improvement in millimetres of mouth opening. Specifically, for mouth opening, negative values support the research hypothesis favouring duloxetine combined with TMJ arthrocentesis for TMJ disorders. Conversely, regarding pain, positive values align with the research hypothesis. The estimated effect sizes for mouth opening closely aligned with the observed effect sizes, indicating minimal variability or heterogeneity between the studies. This consistency suggests that the combination therapy reliably enhances maximal mouth opening, reinforcing its potential efficacy in clinical practice. However, the forest plot analysis and pooled data from both random and fixed effects models revealed that the upper credible limits crossed the line of no effect, −0.34 (−0.79 to 0.11), with an I^2^ value of 27.09%, indicating some amount of insignificance in the effect of the combination treatment on mouth opening (Table 4, Fig. 4b). Consequently, while the reduction in pain with duloxetine was clearly significant, the improvement in maximal mouth opening, although observed, was not conclusively significant.

### Sequential bayes factor analysis

A sequential analysis Bayes factor effect size plot is a visualization used in Bayesian meta-analysis which helps us to understand the strength of evidence for a research hypothesis (H1) compared to a null hypothesis (H0) as the sequential analysis progresses.

- H1: Duloxetine combined with TMJ arthrocentesis is more effective than standalone TMJ arthrocentesis therapy in reducing pain and improvement in mouth opening.
- H0: Duloxetine combined with TMJ arthrocentesis is not more effective than stand-alone TMJ arthrocentesis in reducing pain and improvement in mouth opening.

**Fig. 5:**
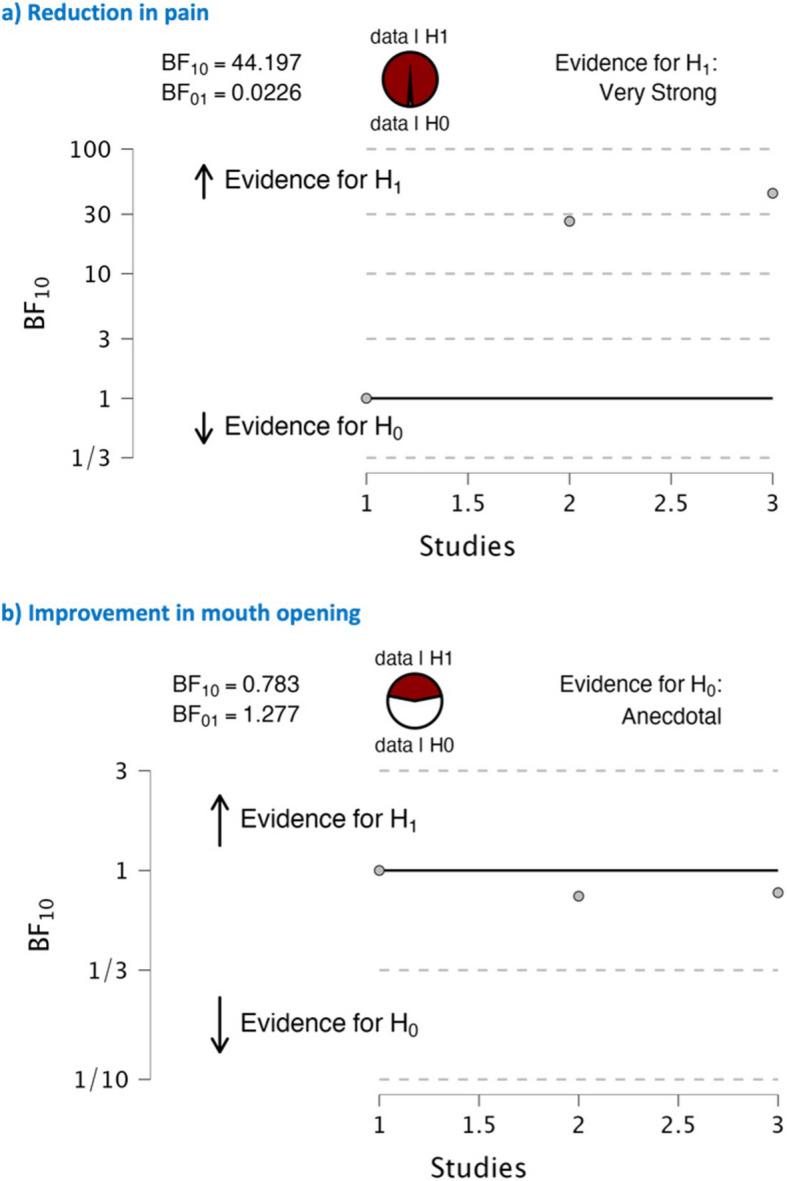
Funnel plot graph on efficiency of combining duloxetine with TMJ arthrocentesis in improvising the parameter of (**a**) pain, (**b**) mouth opening

The Bayes factor (BF), BF₁₀ = 44.197 in the pain parameter indicates that the data is 44.197 times more likely under the alternative hypothesis (H₁) than under the null hypothesis (H₀). Then BF₀₁, the reciprocal of BF₁₀ (i.e., BF₀₁ = 1/BF₁₀), indicates that the data is only 0.0226 times as likely under H₀ as under H₁. In Bayesian terms, a BF₁₀ between 10 and 100 is typically considered'very strong evidence'against the null hypothesis, meaning there is substantial support for the effectiveness of duloxetine combination over standalone TMJ arthrocentesis therapy in reducing pain (Fig. 5a). However, this does not meet the conventional threshold (BF > 100) for'decisive evidence'.

In the case of the evaluation parameter of improvement in maximum mouth opening, BF₁₀ = 0.783 and BF₀₁ = 1.277 suggest that the evidence is weakly in favour of the null hypothesis. This means that there is a slight indication that there is no difference between duloxetine and TMJ arthrocentesis in terms of improvement in mouth opening. However, because the Bayes factor is relatively close to 1 (either way), the evidence is not strong. This situation can be described as inconclusive or weak evidence, indicating that additional data may be required to draw a definitive conclusion regarding the effectiveness of duloxetine combination treatment compared to standalone TMJ arthrocentesis for improving mouth opening (Fig. 5b).

### Certainty of evidence

An evidence profile was generated using the GRADE (Grading of Recommendations Assessment, Development and Evaluation) framework to assess the strength and value of evidence regarding the efficacy of duloxetine in combination with TMJ arthrocentesis and duloxetine with placebo for treating TMDs. This comprehensive assessment involved three randomized controlled trials (RCTs) focusing on key outcomes: pain reduction and improvement in maximum mouth opening. Upon a thorough GRADE assessment, the evidence derived from these RCTs suggests a high level of certainty. Although the studies exhibited a moderate degree of methodological rigor, they consistently and directly addressed the targeted parameters. Moreover, they provided precise and reliable estimates of the intervention's effects. Consequently, the generated evidence strongly supports the clinical utilization of duloxetine alongside TMJ arthrocentesis for managing TMD, instilling a high degree of confidence in both its validity and applicability (Table 5).

**Table 5:** GRADE Summary of findings table for efficacy of duloxetine with TMJ arthrocentesis for TMJ disorders

Certainty assessment							Number of patients		Effect		Certainty	Importance
No. of studies	Study design	Risk of bias	Inconsistency	Indirectness	Imprecision	Other considerations	Duloxetine with TMJ arthrocentesis	TMJ arthrocentesis alone	Relative (95% CI)	Absolute (95% CI)		
**Reduction in pain (assessed with: VAS/NRS (1–10) scale)**												
3 [9, 21, 22]	Randomised trials	serious^a^	not serious	not serious	not serious	all plausible residual confounding would reduce the demonstrated effect	39	39	-	Mean **1.42 higher** (0.74 higher to 2 higher)	⨁⨁⨁⨁ High	CRITICAL
**Improvement in Maximum mouth opening (assessed with: millimetres)**												
3 [9, 21, 22]	Randomised trials	serious	not serious	not serious	not serious	all plausible residual confounding would reduce the demonstrated effect	39	39	-	Mean **0.34 lower** (0.79 lower to 0.11 higher)	⨁⨁⨁⨁ High	IMPORTANT

Explanationsa. Due to issues in random sequence generation and outcome assessment

Inference[31]:High certainty:we are very confident that the true effect lies close to that of the estimate of the effectModerate certainty:we are moderately confident in the effect estimate: the true effect is likely to be close to the estimate of the effect, but there is a possibility that it is substantially differentLow certainty:our confidence in the effect estimate is limited: the true effect may be substantially different from the estimate of the effectVery low certainty:we have very little confidence in the effect estimate: the true effect is likely to be substantially different from the estimate of effect

*CI* Confidence interval

## Discussion

This comprehensive review is centered on the pharmacological interventions employed in the treatment of temporomandibular joint disorders. It further explores the efficacy of duloxetine in combination with the most prevalent management strategies, namely, TMJ arthrocentesis and placebo treatments, in alleviating the associated symptoms. TMD represents a multifaceted condition that significantly impacts the quality of life of affected individuals, necessitating the exploration of novel therapeutic strategies. Our systematic review and Bayesian meta-analysis aimed to critically evaluate the efficacy of duloxetine, a serotonin-norepinephrine reuptake inhibitor (SNRI), in managing TMD symptoms. Our findings suggest that duloxetine, particularly when used in conjunction with TMJ arthrocentesis [9, 17, 21, 22], may offer a valuable therapeutic option for TMD patients, although the evidence supporting its standalone efficacy compared to placebo remains limited [16]. The predominant symptom associated with temporomandibular joint disorders is pain. Our analysis revealed that duloxetine, when combined with TMJ arthrocentesis, demonstrated a medium-to-large effect size in reducing pain. This finding is particularly relevant considering the complex neurobiological mechanisms underlying TMD, which involve aberrant pain processing and mood dysregulation. However, the study comparing duloxetine to placebo did not show a statistically significant reduction in pain, highlighting the need for further research to delineate the contexts in which duloxetine is most effective. The inconsistency in pain reduction outcomes suggests that duloxetine's benefits may be more pronounced when used as part of a multimodal treatment strategy rather than as a standalone therapy. This aligns with findings from studies on chronic pain conditions with a neuropathic component, such as fibromyalgia, where duloxetine has shown efficacy [24]. This finding was further supported by a systematic review [25] that directly compared duloxetine and amitriptyline in the treatment of TMD patients and concluded that both medications demonstrated similar efficacy and safety based on TMD pain rating scales. The study reported comparable effectiveness, with pain reduction exceeding 50% in duloxetine-treated patients and 45% in those receiving amitriptyline, as observed across various controlled trials.

Improvement in maximal mouth opening is another critical outcome for TMD patients, as restricted mandibular movement significantly impairs daily activities and quality of life. Our Bayesian meta-analysis indicated no significant difference in maximal mouth opening between patients treated with duloxetine and those undergoing TMJ arthrocentesis. This finding suggests that while duloxetine may help alleviate pain, it does not significantly enhance functional outcomes related to jaw mobility. The modest effect size for maximal mouth opening improvement underscores the need for adjunctive treatments to address the mechanical aspects of TMD.

The psychological burden of TMD, including depression and anxiety, is well-documented [26]. Duloxetine, with its antidepressant properties, may offer additional benefits by addressing these comorbid psychological symptoms [10]. Our review found that patients reported improved quality of life and reduced symptom severity when treated with duloxetine, particularly in combination with TMJ arthrocentesis. This suggests that duloxetine's role in TMD management may extend beyond pain relief to encompass broader psychosocial benefits, thereby improving overall patient outcomes. The included studies exhibited varying levels of risk of bias, with two studies showing high risk primarily due to issues in random sequence generation and outcome assessment. Despite these concerns, the overall risk of bias was assessed as moderate to high, indicating that the findings should be interpreted with some caution. The GRADE assessment provided high certainty of evidence for the combination of duloxetine and TMJ arthrocentesis, reinforcing the robustness of our conclusions for this specific intervention.

### Adverse effects of duloxetine

A Cochrane systematic review encompassing 18 clinical trials assessed the adverse events associated with duloxetine for neuropathy, chronic pain, or fibromyalgia [24]. The findings indicate that serious adverse events were infrequent and occurred with similar frequency in both duloxetine-treated participants and those receiving a placebo (42 events in 2785 duloxetine participants versus 39 events in 2191 placebo participants; RR 0.81, 95% CI 0.53 to 1.25). This suggests a favourable safety profile for duloxetine, comparable to placebo regarding serious adverse events. The adverse events reported, though rare, included nausea, dry mouth, dizziness, somnolence, fatigue, insomnia, constipation, decreased appetite, sweating, and rhinitis. These adverse events demonstrated a dose-dependent increase in frequency. Notably, at the commonly used dose of 60 mg/day, the incidence of these adverse events remained low. Furthermore, no suicides were reported in the trials where this outcome was assessed, reinforcing the safety of duloxetine in this context.

#### Relation with bruxism

The relationship between bruxism and TMD remains complex and not fully understood. However, studies have reported a significant association, with bruxism identified as a potential risk factor for TMD-related pain. This association appears stronger for myofascial pain and muscle disorders than for disc displacement and intra-articular joint pathologies [29]. A meta-analysis estimated an overall odds ratio of 5.66 (95% CI: 4.63–6.91), highlighting a substantial correlation between bruxism and TMD [30]. Duloxetine has been reported to induce sleep bruxism as a rare side effect [27]. Sahin Onat and Malas [28] described a case in which bruxism developed at 60 mg/day and persisted despite dose reduction to 30 mg/day, resolving only after amitriptyline was added. These findings indicate that the mechanisms underlying SNRI-induced bruxism remain unclear, highlighting the need for careful monitoring of bruxism symptoms when prescribing duloxetine.

### Limitations and future research

The findings of our systematic review hold significant clinical implications. Duloxetine, especially when used alongside TMJ arthrocentesis, shows promise in managing TMDs, particularly in patients dealing with both pain and psychological symptoms. However, we couldn't definitively establish duloxetine's effectiveness compared to a placebo when used alone, so further research is necessary. The limited number of studies and participants emphasizes the need for more research to confirm these initial findings and understand duloxetine's long-term effects and safety in TMD treatment. Future studies should focus on large-scale, well-designed trials to determine the best way to use duloxetine in managing TMD. Understanding why people respond differently to duloxetine and how it fits into broader treatment plans could greatly improve patient outcomes. Exploring how duloxetine could work together with other treatments like physical therapy and cognitive-behavioural therapy could also provide valuable insights. In summary, our findings suggest that including duloxetine as part of a comprehensive treatment plan for TMD could be beneficial, but more research is needed to fully understand its role and optimize patient care.

## Conclusion

Despite variations in study designs and treatment modalities, the collective findings suggest that duloxetine, particularly when combined with TMJ arthrocentesis, may offer significant pain relief and improvement in functional outcomes for TMD patients. However, the interpretation of these findings should be tempered by the moderate-to-high risk of bias observed across the included studies, underscoring the need for further well-designed randomized controlled trials to confirm the preliminary evidence and elucidate duloxetine's long-term efficacy and safety profile in TMD management.

## Data Availability

All data supporting the findings of this study are available within the paper.

## Abbreviations

TMD: Temporomandibular Joint Disorder
NSAID: Non-Steroidal Anti-Inflammatory Drugs
SNRI: Serotonin-Norepinephrine Reuptake Inhibitor
SD: Standard Deviation
MCMC: Markov Chain Monte Carlo
GCPS: Graded Chronic Pain Scale
VAS: Visual Analogue Scale
NRS: Numerical pain Rating Scale
HADS: Hospital Anxiety and Depression Rating Scale
PHQ: Patient Health Questionnaire
MMO: Maximum Mouth Opening
SM: Self-Management
GB: Group B
GC: Group C
